# Improvement of Adolescent Idiopathic Scoliosis Primary Correction by Brace Design Optimization

**DOI:** 10.3390/children9050656

**Published:** 2022-05-03

**Authors:** Patrick Strube, Chris Lindemann, Max Bahrke, Steffen Brodt, André Sachse, Lya I. Reich, Alexander Hoelzl, Timo K. Zippelius

**Affiliations:** 1 Orthopedic Department, Jena University Hospital, Campus Eisenberg, Klosterlausnitzer Str. 81, 07607 Eisenberg, Germany; c.lindemann@waldkliniken-eisenberg.de (C.L.); maxbahrke@gmx.de (M.B.); s.brodt@waldkliniken-eisenberg.de (S.B.); a.sachse@waldkliniken-eisenberg.de (A.S.); a.hoelzl@waldkliniken-eisenberg.de (A.H.); 2 Department of Orthopedic Surgery, University Hospital Ulm, Oberer Eselsberg 45, 89081 Ulm, Germany; lyaimke.reich@rku.de (L.I.R.); timo.zippelius@rku.de (T.K.Z.)

**Keywords:** bracing, idiopathic scoliosis, conservative treatment, brace type, cheneau brace, primary correction, Cobb angle, apex rotation

## Abstract

(1) Background: Primary in-brace correction has been shown to be related to conservative adolescent idiopathic scoliosis (AIS) treatment outcome. The purpose of the study was to evaluate TLSO brace design changes over eight years regarding primary Cobb correction and de-rotation of the (major) curve. (2) Methods: This retrospective analysis included AIS patients treated with a full-time TLSO-brace in a single Orthopedic University hospital in 2012–2014 and 2017–2019. Brace design changes resulted from an evolutionary process, from a 3-point bending to a 3D TLSO. The brace parameters (presence of an anterior shoulder pad, posterior high-thoracic pad, thoracic space, and angle of the lumbar and thoracic pressure points) were analyzed regarding the primary (major) Cobb angle and apex rotation correction with a multivariate analysis. (3) Results: A total of 74 (63 female) patients were included in the study. The Cobb angle primary correction was significantly improved over the timeframe. The primary correction was significantly influenced by all design parameters and partially by its interactions with (curve specific) different effects on the Cobb correction and apex de-rotation. (4) Conclusions: Knowledge about the effects of brace design parameters on the curve’s angle and rotation correction enables improvements in individualized brace design and the brace optimization process.

## 1. Introduction

Corrective brace wear is a long-established treatment option for patients with idiopathic scoliosis [1,2,3]. In around 70% of cases, progression of curvature can be halted and ultimately surgery can be avoided [1,2]. However, this also means that despite the proven effectiveness of brace treatment, the failure rate ranges between 12 and 39% [3]. Besides patients’ compliance in wearing the brace, the known factors influencing the success of conservative treatment are location (main thoracic vs. lumbar), flexibility, symmetry and magnitude of the curve, degree of apical rotation, lateral deviation of the apex from the midline, and the magnitude of the initial correction of the rotation and curve [4,5,6,7,8]. The latter is mainly dependent either on curve flexibility or on the brace’s ability to address these parameters, which is strongly related to brace design. To better compare conservative treatments, the International Society on Scoliosis Orthopedic and Rehabilitation Treatment (SOSORT) recently published a new classification system for brace designs [9]. This classification was based on several parameters: primary effect (bending, detorsion, elongation, movement, push-up, three points), rigidity (very rigid, rigid, elastic), localization (cervical—C, thoracic—T, lumbar—L: CTLSO, TLSO, LSO, whereas SO means sacral orthosis), primary plane of correction (frontal, sagittal, transversal, frontal and sagittal, frontal and transversal, sagittal and transversal, three-dimensional), and construction (valves: monocot, bivalve, multisegmented; closure: posterior, lateral, anterior). Nevertheless, many different brace designs exist and outcome as well as primary curve correction effects are heterogenous [10]. Almost nothing is known regarding the modification of different design parameters of the braces and its effect on primary rotation and curve correction. This might be of special importance because most of braces are individually designed and manufactured.

Therefore, the aim of the present study was to analyze the effect of different brace design modifications, performed over eight years of brace manufacturing, on the primary curve correction by a rigid, three-dimensionally acting TLSO.

## 2. Patients and Methods

### 2.1. Inclusion and Exclusion Criteria

**Figure 1:**
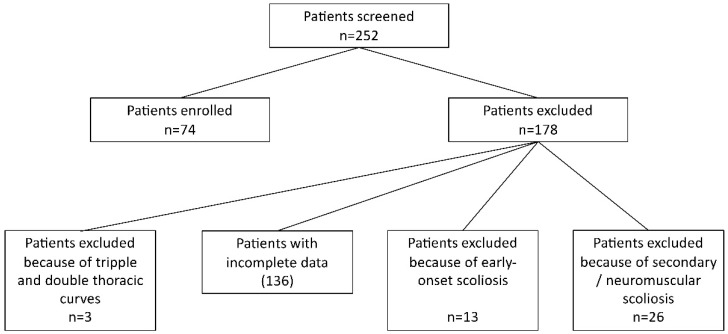
The flow chart of in/exclusion process.

Screening was performed in conservatively treated scoliosis patients at a single Orthopedic University department (Figure 1). The inclusion criteria were: patients with single or double curve AIS, at least 10 years of age, Risser stage 0–2, the curve had to have been between ≧20° and <50° (an extension of the inclusion criteria compared to the SOSORT-SRS consensus in 2014 because of the rigid treatment approach) at the beginning of treatment, treatment with a full-time TLSO brace and scoliosis-specific exercises according to Schroth [11,12]. The brace had to be manufactured from 2012 to 2014 (for early models of the brace designs) or from 2017 to 2019 (for recent models of the brace designs). Further inclusion criteria consisted of the presence of whole-spine anterior-posterior radiographs before and after brace fitting as well as the presence of a three-dimensional computer-aided-design/computer-aided-manufacturing (CAD/CAM) model of the brace used. Patients with incomplete data and those with secondary scoliosis or congenital scoliosis were excluded, as were double thoracic or triple curves.

The study was conducted in accordance with the Declaration of Helsinki, and the protocol was approved by the Ethics Committee of Jena University Hospital (Project identification code 2018-1098) and approved on 23 July 2018.

### 2.2. Brace Design and Modifications

**Figure 2:**
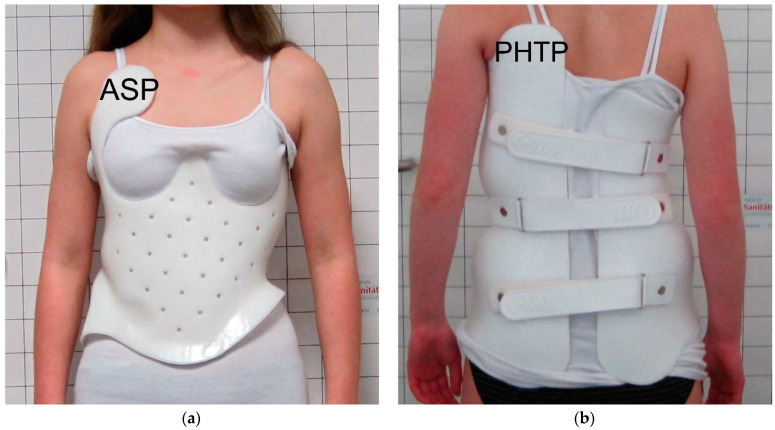

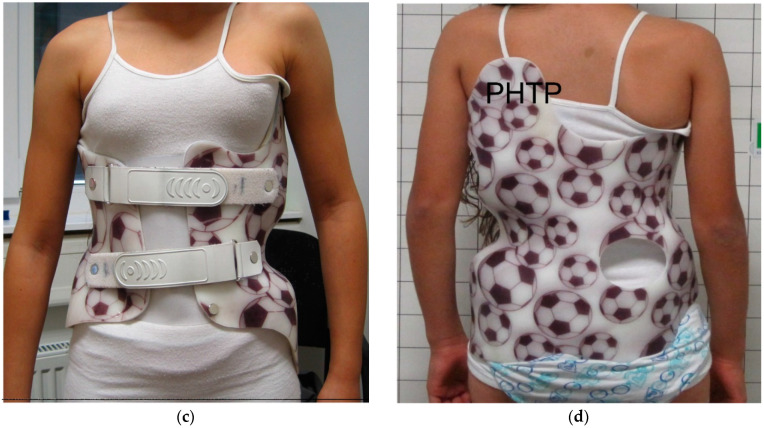
Examples of early and recent braces: (**a**) anterior view of an early brace from 2012, ASP marks the anterior shoulder pad; (**b**) posterior view of the same brace, PHTP marks the posterior high-thoracic pad position; (**c**) anterior view of a recent brace from 2019, (**d**) posterior view of this brace including PHTP. ASP: anterior shoulder pad. PHTP: posterior high thoracic pad.

All patients were fitted with a full-time TLSO brace (SOSORT scoliosis brace classification [9]: early: TLSO rigid, 3-point bending, frontal and transverse, monocot with posterior closure; recent: Cheneau-like TLSO, rigid, detorsion, 3D, monocot with ventral closure, Figure 2c,d) from the hospital’s own manufacturer and were asked to wear the brace for 23 h per day. The patients underwent three-dimensional scanning with a Spectra^TM^ (Vorum, Vancouver, BC, Canada) or M4D scan (Rodin GmbH, Gerstetten-Heldenfingen, Germany, since 2013), and the scan was matched with the radiographs. A virtual brace model was created with the modeling/processing software Canfit^TM^-Plus (Vorum) or Rodin4D (Rodin GmbH, since 2013). According to this, the patient’s brace was produced and then optimized regarding fitting as required. After wearing the brace for four to six weeks, repeat in-brace radiographs were reviewed by a physician and the brace was re-evaluated by an orthotist. From 2012 to 2019, a continuous evolutionary and modulatory process was undertaken by the manufacturer to further optimize brace effectiveness. A consistent group of experienced orthotists was involved in the design and manufacturing over time. This led to the following qualitative modifications to the brace design:

- Depending on the curve localization, a high thoracic (shoulder) pad was added posteriorly (no anterior shoulder pads were placed in newer braces), in earlier braces it was often placed anteriorly or anteriorly and contralaterally posteriorly;
- The thoracic pressure point at the posterior rib hump was pronounced but was shifted from a lateral to a more posterolateral position;
- Thoracic space was increased anterolaterally at the convex side to allow more active correction here;
- The lumbar pressure point was shifted from a posterolateral to a more posterior position;
- All pressure pads were designed to be flatter;
- Closure was changed from posterior to anterior.

As described above, the process was evolutionary. Therefore, and because of the individuality of the curves and braces, not every new brace includes all modifications. Figure 2 illustrates the design modifications from 2012 to 2019. The two timeframes were chosen to provide a good contrast between the new and old brace designs.

### 2.3. Brace Model Measurements

**Figure 3:**
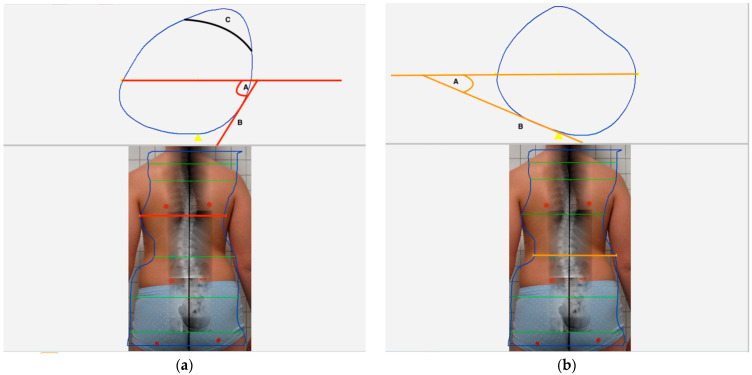
The figure shows the measurement technique of the lumbar (LPA) and the thoracic pressure point angles (TPA) as well as the position of the thoracic space. (**a**) The lower part of this image shows the level of the measurement plane (orange) at the lumbar apex. The a.p. X-ray image was transposed to the posterior photographic view of the patient and the planned 3-dimensional computer-aided-design – computer-aided-manufacturing (CAD-CAM) model of the brace was superposed before. The upper part illustrates the cross-sectional area of the brace model at this orange level. A tangent was drawn to the lumbar pressure point ‘B’ and the angle between this tangent and the transversal body axis ‘A’ was measured = LPA. (**b**) The lower part of this image shows the level of the measurement plane (orange) at the thoracic apex. The upper part illustrates the cross-sectional area of the brace model at this orange level. A tangent was drawn to the thoracic pressure point ‘B’ and the angle between this tangent and the transversal body axis ‘A’ was measured = TPA. ‘C’ shows the usual position of the thoracic space (TS).

- The following parameters were measured in the 3D-software model of each brace (Figure 3a,b):
- Presence of an anterior shoulder pressure pad contralateral to the thoracic pad (ASP yes/no);
- Presence of a posterior high thoracic (shoulder) pressure pad contralateral to the thoracic pad (PHTP yes/no);
- Built-in thoracic space (TS—yes/no);
- Angle of the thoracic pressure point (TPA) in relation to the transversal body/brace axis (°);
- Angle of the lumbar pressure point (LPA) in relation to the transversal body/brace axis (°).

### 2.4. Radiographic Measurements

The DICOM (Digital Imaging and Communications in Medicine) radiographs obtained before and at initial control (in-brace) were sourced from the local PACS (Picture Archiving and Communication System), magnified, and evaluated. The Cobb angle and the rotation of the apical vertebra (according to Nash and Moe) [13] for every (structural) curve were measured. Apex rotation was more precisely determined by measuring the distance from the center of the convex-sided pedicle to the convex-sided edge of the vertebra in mm. To compensate for potential errors regarding individual vertebral size, this distance was related to the width of the apex vertebra (distance between left and right edge of the apical vertebra) measured in mm: NaM. In double-curve patients, the measurements were performed separately and evaluated accordingly (Cobb1, Cobb2, NaM1, NaM2). The larger of the two angles was designated Cobb1 = major curve, the smaller angle Cobb2 = minor curve. NaM1 therefore means apical rotation of the major and NaM2 that of the minor curve. Delta values were calculated between pre-treatment and in-brace Cobb and NaM measurements. The correction rate (Cr) was also calculated for all six parameters = 100 − (in-brace parameter/pre-treatment parameter * 100). Apex localization (APL) of the (major) curve thoracic (T)/lumbar (L)/thoracolumbar (TL) was also assessed on the pre-treatment images. Two raters (M.B., C.L.) analyzed the radiographs, classified them as single or double curve, and measured the parameters independently and blinded. The mean values were used for data analysis of the continuous parameters and in the case of conflicting results in categorical parameters, the decision was made by a third independent rater (P.S.).

### 2.5. Clinical Data

Sex and age at outset of treatment were documented for analysis. Additionally, the clinical–radiological failure rate of the conservative treatment was determined from the patient files (rate of patients who required surgery or missed the <45° major Cobb angle threshold 6 months after weaning from the brace at Risser stage ≥4).

### 2.6. Statistical Data Analysis

Statistical analysis was performed using SPSS^®^ Statistics Version 28.0 (IBM, Armonk, NY, USA).

The changes in the brace parameters were exploratively analyzed to display changes over time and the early and the recent timeframes were compared with the Student’s *t*-test and Fisher’s exact test. Additionally, apex location, age, and brace parameters (ASP, PHTP, TS, TPA, LPA) were implemented in a multivariate linear model to determine the influence on DeltaCobb1, DeltaCobb2, on DeltaNaM1 and DeltaNaM2, and the on all parameters’ correction rates in single and double curves. Post hoc tests to further discriminate significant effects were performed with one- or two-way ANOVA (analysis of variance) and post hoc Bonferroni tests or 2-sided Student’s *t*-tests. A Kolmogorov–Smirnov test was performed before applying parametrical tests. The conservative treatment failure rate between early and recent braces was compared with Fisher’s exact test. The level of significance was set to *p* < 0.05.

## 3. Results

### 3.1. Clinical and Radiographic Results

A total of 74 patients were identified who fulfilled the inclusion criteria and could participate in the study. Table 1 shows the patient characteristics and radiographic measurements of the patients as well as the differences regarding the early and recent timeframes. The mean Cobb1 and Cobb2 correction rates were 35.3% and 21.6%, respectively, and were improved from 31.1 to 38.7% (Cobb1, *p* = 0.152) and from 10.7 to 30.5% (Cobb2, *p* = 0.026) from the early to the recent timeframe. The differences in the rotation parameter between the timeframes were much smaller and did not reach significance. The conservative treatment failure rate improved non-significantly (*p* = 0.246) from 27.3 % (early braces) to 14.6 % (recent braces) (Table 1).

### 3.2. Brace Parameter Changes over Time

**Figure 4:**
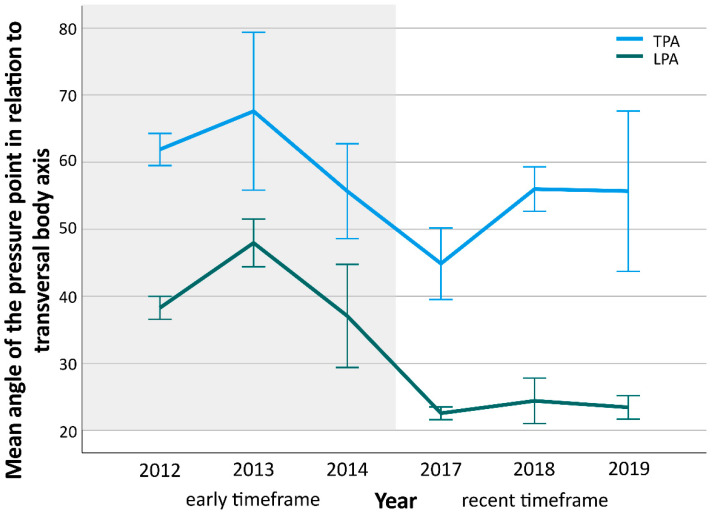
The diagram shows the distribution of mean angles of the lumbar (LPA) and thoracic pressure point angle (TPA) over time. Early timeframe is marked with gray, recent with white background. Mean angle differences between both timeframes were significant (TPA *p* = 0.011; LPA *p* < 0.001). Whiskers indicate single standard error.

The modifications of the position of the thoracic (TPA, early = 60.9°, standard deviation (SD) = 15.7°, recent = 50.8°; SD = 21.2°) and the lumbar pressure point (LPA, early = 39.1°, SD = 13.6°, recent = 23.4°, SD = 8.9°) led to significant (TPA *p* = 0.011; LPA *p* < 0.001) differences between the timeframes (Figure 4). Additionally, all qualitative parameter changes (ASP, PHTP, and TS) were significant (all *p* < 0.001) between early and recent timeframe (Table 2).

### 3.3. Results of Multivariate Analysis

The sub-analysis of the 20 patients with double curves did not show any significant effects on major or minor curves regarding Cobb reduction (DeltaCobb1/2/CrCobb1/2) or apex de-rotation (DeltaNaM1/2/CrNaM1/2). Therefore, analysis was focused on the major curves of all patients.

The significant main effects on the Cobb angle or apex rotation correction from multivariate analysis are indicated in Table 3. These significant main effects are descriptively explained in the following paragraphs. Therefore, all subsequent p-values represent that of post hoc tests.

#### 3.3.1. Patient Parameters

**Figure 5:**
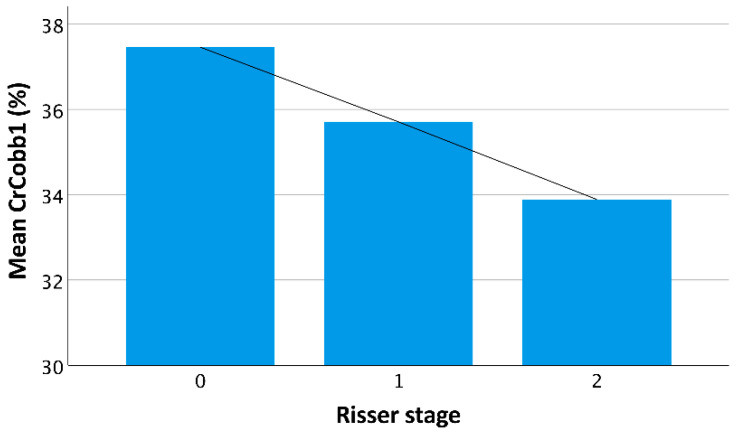
The diagram illustrates the mean Cobb angle correction rate by the braces in relation to patients’ Risser stage. The line illustrates the decrease in mean correction with increasing skeletal maturity (*p* = 0.858).

According to the main effects, we found that an increase in age led to a moderate decrease in Cobb1 correction (post hoc DeltaCobb1 *p* = 0.371, CrCobb1 *p* = 0.145). Higher Risser stage led to inferior primary Cobb angle correction (Figure 5), but the effects of Risser stage on angle and rotation correction were not significant (DeltaCobb1 *p* = 0.480, CrCobb1 *p* = 0.858, DeltaNaM1 *p* = 0.488, CrNaM1 *p* = 0.543). Post hoc tests revealed the best angle and (not significantly) rotational correction of (major) curves with thoracolumbar apex (CrCobb1: T 30.2% SD 22%, L 36.9% SD 22.7%, TL 52.6% SD 17.7%, *p* = 0.023, T vs. TL *p* = 0.021; DeltaNaM1: T 0.015 SD 0.042, L 0.019 SD 0.042, TL 0.050 SD 0.045, *p* = 0.085; CrNaM1: T 4.6% SD 14.9%, L 6.8% SD 15.2%, TL 14.9% SD 12.3%, *p* = 0.175).

#### 3.3.2. Brace Parameters

**Figure 6:**
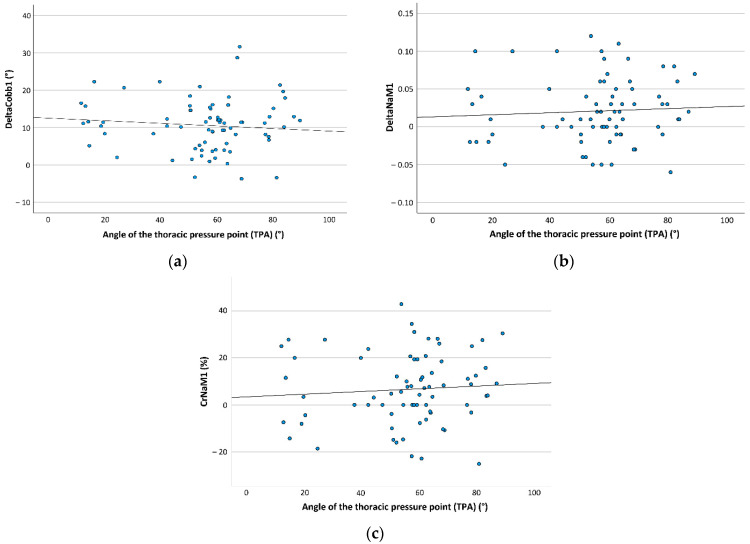
The figure demonstrates the distribution of mean angular Cobb (DeltaCobb1) and apex rotation correction (DeltaNaM1; correction rate CrNaM1) in relation to the angle of the thoracic pressure point angle (TPA), which were significant in multivariate general linear model (DeltaCobb1 *p* = 0.028, DeltaNaM1 *p* = 0.037, CrNaM1 *p* = 0.016). (**a**) The diagram shows TPA distribution in relation to DeltaCobb1. The black interpolated line demonstrates that with decreasing TPA, DeltaCobb1 increased. (**b**) The diagram shows TPA distribution in relation to DeltaNaM1. The black interpolated line demonstrates that with decreasing TPA, DeltaNaM1 decreased. (**c**) The diagram shows TPA distribution in relation to CrNaM1. The black interpolated line demonstrates that with decreasing TPA, CrNaM1 decreased.

**Figure 7:**
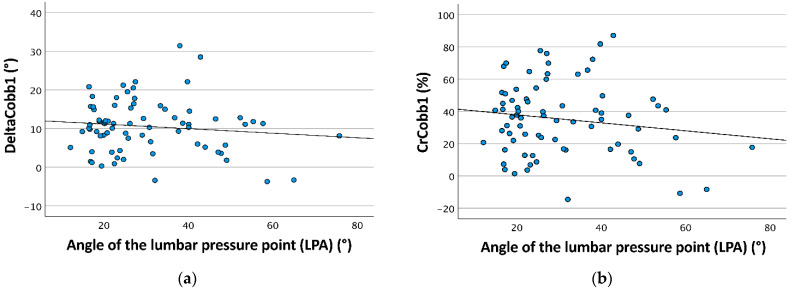
The figure demonstrates the distribution of the mean angular Cobb correction (DeltaCobb1) and mean Cobb angle correction rate (CrCobb1) in relation to the angle of the lumbar pressure point angle (LPA), which were significant in the multivariate general linear model (DeltaCobb1 *p* = 0.030, CrCobb1 *p* = 0.024). (**a**) The diagram shows LPA distribution in relation to DeltaCobb1. The black interpolated line demonstrates that with decreasing LPA, DeltaCobb1 increased. (**b**) The diagram shows LPA distribution in relation to CrCobb1. The black interpolated line demonstrates that with decreasing LPA, CrCobb1 increased.

The presence of the anterior shoulder pad led to better de-rotation of the (major) curve’s apex (DeltaNaM1: ASP(+) 0.027, SD 0.045; ASP(−) 0.018, SD 0.042; CrNaM1: ASP(+) 8.5%, SD 14.6%; ASP(−) 5.9%, SD 15.1%). Regarding the thoracic and lumbar pressure point angle, Cobb correction was better in smaller angles (Figure 6a and Figure 7). Conversely, a smaller TPA resulted in less de-rotation of the apex (Figure 6b,c).

#### 3.3.3. Interaction of Patient and Brace Parameters

Because all post hoc tests in the subsequent analysis (Section 3.3.3 and Section 3.3.4) were not significant, the differences must be judged as descriptive only to better understand the direction of the significant main effect revealed in multivariate analysis.

The presence of the anterior shoulder pad (ASP(+)) resulted in the largest de-rotation in TL (major) curves, whereas ASP(+) led to better de-rotation in thoracic and thoracolumbar apex locations and to smaller de-rotation in lumbar apex location curves; see Table 4.

PHTP presence was associated with the largest Cobb angle correction in T-curves followed by TL-curves (about half of T-correction), and L-curves (no improvement effect/slight loss of correction). The absence of PHTP in T-curves led to almost no mean angle correction. Regarding de-rotation, PHTP presence was positively associated with T- and TL-curves but a slightly negatively with L-curves. The absence of PHTP had a negative rotational association with T-curves (Table 4).

The addition of thoracic space resulted in improved Cobb angle correction in TL-curves only, whereas in T- and especially in L-curves, a slight decrease in correction was observed. Thoracic space also resulted in larger de-rotation in TL-curves than in T-curves, but in L-curves, reduced de-rotation could be observed if TS was present (Table 4).

#### 3.3.4. Interaction of Brace Parameters

Regarding the interaction of thoracic space and the anterior shoulder pad, the best Cobb correction could be observed if both were not present, followed by ASP(−) and TS(+). The presence of both brace design modifications resulted in the lowest Cobb angle correction (Table 5). In contrast to this, thoracic space was associated with better de-rotation regardless of whether ASP was present or not. Adding ASP could have an additional positive effect on de-rotation if thoracic space was present (Table 5).

Better Cobb correction could be observed in braces with PHTP and without ASP and vice versa. De-rotation was larger with ASP in braces without PHTP and smaller in braces without any of both pressure pads (Table 5).

## 4. Discussion

The aim of the present study was to analyze the effect of different brace design modifications, performed over eight years of brace manufacturing, on the primary curve correction by a rigid, three-dimensionally acting TLSO. We found that most of the modifications (to curve-type, specifically) influence the primary correction of the (major) curve’s Cobb angle and its apex rotation in AIS patients.

First, we could improve the Cobb angle correction from about 30 to 39% for major curve (and from 11 to 31% for minor curve in cases with double curves). However, this improvement was only significant for the secondary curve. Therefore, in general, the brace design modifications performed can be judged as positive. Based on the design parameter findings of the multivariate analysis, further improvement seems to be possible in the future. The Cobb angle correction rate is in the middle range of those reported in the literature. Depending on the brace type, primary correction rates of 10 to 60% can be found [7,14,15,16]. In a review, Zaina et al. reported a primary correction rate of 31 to 41% for Cheneau-like braces, which are similar to our recent brace models [10]. Age had an influence on primary Cobb correction in our cohort. This result is in line with that of Weiss [17]. However, other authors did not find such an association [18]. The conservative treatment failure rate also (non-significantly) decreased from early (27.3%) to recent (14.6%) models and was d within the range of published failure rates for both [3,4]. Nevertheless, failure rate is also dependent on other parameters that were not analyzed here (e.g., compliance).

The analysis of both pressure point angles (lumbar and thoracic) showed that a smaller angle led to a better (major) Cobb correction. However, in case of TPA, the shift to a more posterolateral position also had a concomitant negative effect on de-rotation. Therefore, the evolutional angle changes performed led to a better Cobb correction, but to an inferior apex de-rotation. Additionally, the presence of an anterior shoulder pad resulted in a better de-rotation of the major curves’ apex. Adding thoracic space seems to further improve this outcome. Nevertheless, Cobb angle correction was negatively associated with the presence of both parameters. Therefore, a compromise must be made regarding implementation of these parameters based on the decision to further address the Cobb angle or the apex rotation correction. With regard to our manufacturing history, TPA was reduced, ASP was less frequently and TS was more frequently built in the braces in the recent timeframe. This suggests that a decision to focus on Cobb angle correction drove this development. Since surgical decision-making is mostly related to Cobb thresholds and because Cobb angle correction is easier to monitor, retrospectively, the historical development seems to be quite pragmatic. Nevertheless, as demonstrated in Figure 4, TPA increased again after 2017. This could be an indication that more attention was paid to apex de-rotation in the most recent timeframe.

The situation becomes a bit more complex when looking at the combined results of posterior high-thoracic and anterior shoulder pad presence. If PHTP was added, the Cobb correction results were better without an anterior shoulder pad, whereas de-rotation was better with ASP instead of a posterior high-thoracic (shoulder) pad. Therefore, again development was driven by a better Cobb correction, while implementing PHTP more often and ASP less frequently. As a compromise, PHTP and TS could somewhat compensate for the lost effects of ASP and TPA on de-rotation. However, it is possible that ASP will be built in more frequently again in future braces, especially in those where a PHTP is not necessary and the apex rotation is large.

The question could arise as to why to implement more thoracic space. Looking at the apex location specific outcome, the answer can be found easily. Adding thoracic space improves the de-rotation of T- and especially TL-curves. Additionally, it improves Cobb correction in TL-curves. Based on the results, in (major) curves with lumbar apex it should be avoided, because it resulted in an inferior outcome. Similarly, PHTP was found to be beneficial in T- and TL-curves regarding Cobb correction and de-rotation. In (major) lumbar curves it was related to a slightly inferior outcome. Therefore, PHTP should be implemented in all T- and TL-curves, as was done in our recent timeframe. With regard to ASP, in L-curves it affects de-rotation negatively although a positive association with de-rotation in T- and TL-curves could be observed, similar to that of PHTP. Because PHTP has an additional positive influence on Cobb correction, PHTP should be preferred to ASP. The evolutionary decisions made regarding brace design support this post hoc conclusion.

Other bracing parameters have been reported to influence primary curve correction. Karam et al. reported a better Cobb correction in X-ray images of fulcrum bending in AIS patients if the fulcrum had been placed more superiorly (at the level of the apex of the curve) [19]. They concluded that a more superior placement of the thoracic pad would be more effective in Cobb correction during bracing. This was confirmed for T-curves by a recent analysis of different TLSO-brace types using finite element modeling (FEM) for brace effect simulation [20]. Besides this, in this FEM study, an angle variation in the pressure points (vectors) was also found to influence correction. However, the sagittal profile was mainly affected here, which was not analyzed in the present study and must be part of future studies. Furthermore, FEM simulations resulted in a small but significant de-rotation if an anterior pad had pushed the rib cage. This observation is comparable to the results regarding anterior shoulder pad outcome.

The present study is not without limitations. First, the analysis only included the results of a single manufacturer and design. Therefore, transferring the results to other brace designs may be difficult and result in different outcomes. Second, the cohort analyzed is rather small. Especially, subgroup analyses become underpowered, for example, the subgroup of double curves in the present study. However, multivariate analysis resulted in significant main effects and allowed the identification of relevant parameters and the direction of their effects. Third, initial curve correction is also a function of curve flexibility. This parameter could not be measured and may represent an unrecognized confounder. Fourth, rotational measurement was based on X-ray images and was performed according to Nash and Moe. Accuracy can further be improved using 3D images or 3D reconstructions [21]. Both were not available in the present study. Fifth, Risser stage is a somewhat unprecise method of describing bone maturity, which could have led to a bias in the inclusion of older individuals (the single 17-year-old patient had a retarded growth spurt because of growth hormone deficiency). Different methods such as tri-radiate cartilage ossification and Sanders stage are better for determining skeletal maturity [22,23] but could not be included because of missing hand X-rays and no imaging of the hips in the total spine X-rays of many patients. Finally, the effects on sagittal profile were not analyzed because lateral images were not available for many of the patients.

## 5. Conclusions

In conclusion, we were able to describe TLSO-brace parameter changes that were associated with improved primary Cobb angle correction and de-rotation of the (major) curve of AIS patients. Based on these findings, individual design decisions can be made more easily, and braces can be improved further. Future studies should address the effects of brace design parameters on sagittal alignment and different brace types.

## Figures and Tables

**Table 1:** Patient characteristics and radiographic measurements.

Parameter		All Patients *n* = 74	Early Group *n* = 33	Recent Group *n* = 41	*p*-Value
mean age (min-max)		14.2 (10–17)	14.1 (10–16)	14.3 (10–17)	0.708 *
sex (f/m)		63/11	28/5	35/6	1.000 ^#^
single/double curves		54/20	24/9	30/11	1.000 ^#^
conservative treatment failure rate		20.3%	27.3%	14.6%	0.246 ^#^
apex of the curve (T/L/TL)		26/39/9	11/19/3	15/20/6	0.680 ^#^
Mean Cobb1 (95% CI) (°)	before treatment	30.4 (27.7–32.1)	30.5 (27.9–30)	30.3 (28.1–32.6)	0.939 *
	in-brace	19.9 (17.9–21.9)	21.2 (17.8–24.5)	18.8 (16.3–21.4)	0.246 *
Mean DeltaCobb1 (95% CI) (°)		10.5 (8.9–12.1)	9.3 (6.6–12)	11.5 (9.6–13.5)	0.162 *
Mean CrCobb1 (%)		35.3 (30–40.6)	31.1 (22.5–39.6)	38.7 (32–45.4)	0.152 *
Mean Cobb2 (95% CI) (°) ^†^	before treatment	30.3 (27.2–33.3)	29.2 (23.4–35.1)	31.1 (27.2–35)	0.273 *
	in-brace	23.4 (20.1–26.8)	25.8 (19.6–32)	21.5 (17.5–25.5)	0.093 *
Mean DeltaCobb2 (95% CI) (°) ^†^		6.8 (3.8–9.8)	3.5 (−1.1–8)	9.6 (5.8–13.3)	0.028 *
Mean CrCobb2 (%) ^†^		21.6 (12–31.1)	10.7 (−5.7–27.1)	30.5 (20.2–40.8)	0.026 *
Mean NaM1 (95% CI)	before treatment	0.28 (0.26–0.29)	0.28 (0.25–0.3)	0.29 (0.26–0.29)	0.798 *
	in-brace	0.26 (0.24–0.27)	0.25 (0.23–0.28)	0.26 (0.24–0.28)	0.633 *
Mean DeltaNaM1 (95% CI)		0.02 (0.01–0.03)	0.02 (0.01–0.04)	0.02 (0.01–0.03)	0.774 *
Mean CrNaM1 (%)		6.6 (3.2–10.1)	7.2 (2–12.3)	6.2 (1.3–11.1)	0.787 *
Mean NaM2 (95% CI) ^†^	before treatment	0.26 (0.24–0.28)	0.26 (0.22–0.29)	0.27 (0.23–0.3)	0.654 *
	in-brace	0.25 (0.21–0.28)	0.25 (0.21–0.29)	0.24 (0.19–0.3)	0.803 *
Mean DeltaNaM2 (95% CI) ^†^		0.02 (0.01–0.04)	0.01 (−0.01–0.02)	0.03 (−0.02–0.07)	0.399 *
Mean CrNaM2 (%) ^†^		6.9 (−1.7–15.4)	3.1 (−4.5–10.7)	9.9 (−5.6–25.4)	0.392 *

* *p*-values from *t*-test; ^#^ *p*-values from Fisher’s exact test; ^†^ values for patients with double curves: *n =* 20 (9 early, 11 recent); CI means confidence interval; T thoracic apex, L lumbar apex, TL thoracolumbar apex.

**Table 2:** Qualitative brace changes.

Parameter		Early Group (*n =* 33)	Recent Group (*n =* 41)	*p*-Value *
ASP	no	13	40	
	yes	20	1	<0.001
PHTP	no	9	0	
	yes	24	41	<0.001
TS	no	21	6	
	yes	12	35	<0.001

ASP—anterior shoulder pad, PHTP—posterior high thoracic pad, TS—thoracic space; * *p*-values from Fisher’s exact test.

**Table 3:** Major curve analysis results (*p*-values).

Parameter	DeltaCobb1	CrCobb1	Effect on (Major) Curve Angle	DeltaNaM1	CrNaM1	Effect on (Major) Curve Rotation
Age	0.008	0.012	yes	0.582	0.309	no
Apex localization (APL)	0.133	0.010	yes	0.003	0.005	yes
Anterior shoulder pad (ASP)	0.456	0.187	no	0.007	0.005	yes
Posterior high-thoracic pad (PHTP)	0.094	0.160	no	0.904	0.983	no
Thoracic Space (TS)	0.868	0.321	no	0.169	0.214	no
Angle of the thoracic pressure point (TPA)	0.020	0.102	yes	0.037	0.016	yes
Angle of the lumbar pressure point (LPA)	0.032	0.006	yes	0.627	0.367	no
APL*ASP	0.220	0.160	no	0.019	0.017	yes
APL*PHTP	0.005	<0.001	yes	0.077	0.045	yes
APL*TS	0.004	0.002	yes	0.034	0.010	yes
ASP*TS	0.004	0.001	yes	0.064	0.024	yes
ASP*PHTP	0.003	<0.001	yes	0.005	0.001	yes
TS*PHTP	0.991	0.850	no	0.537	0.527	no

All *p*-values are from multivariate linear model. Triple or higher combinations of factors did not lead to significance.

**Table 4:** Apex-specific brace parameter effects on (major) curve.

Apex Location		ASP(+)	ASP(−)	PHTP(+)	PHTP(−)	TS(+)	TS(−)
T	Mean DeltaCobb1 (SD)	n.s.	n.s.	10.6 (7.4) °	0.1 (4.9) °	10 (6.9) °	10.3 (10) °
	Mean CrCobb1 (SD)	n.s.	n.s.	31.8 (21.1) %	0.7 (21.7) %	29.3 (17.6) %	33 (32.6) %
	Mean DeltaNaM1 (SD)	0.026 (0.042)	0.007 (0.041)	n.s.	n.s.	0.017 (0.039)	0.011 (0.050)
	Mean CrNaM1 (SD)	8.9 (13.8) %	1.6 (15.2) %	5.1 (14.4) %	−5.7 (27.3) %	4.9 (14.3) %	3.7 (17.3) %
L	Mean DeltaCobb1 (SD)	n.s.	n.s.	9.9 (6.5) °	11 (3.3) °	9.4 (6.3) °	11 (5.5) °
	Mean CrCobb1 (SD)	n.s.	n.s.	35 (23.6) %	43.4 (20) %	32.1 (21.1) %	42.6 (24.2) %
	Mean DeltaNaM1 (SD)	0.010 (0.050)	0.021 (0.041)	n.s.	n.s.	0.019 (0.048)	0.020 (0.036)
	Mean CrNaM1 (SD)	2.3 (16.9) %	7.6 (15.2) %	6.3 (16.7) %	8.7 (10) %	5.9 (16.7) %	7.8 (14) %
TL	Mean DeltaCobb1 (SD)	n.s.	n.s.	14.3 (6.1) °	9.2 (n.a.) °	17.4 (4.8) °	10.8 (5.3) °
	Mean CrCobb1 (SD)	n.s.	n.s.	54.1 (18.3) %	40.7 (n.a.) %	63.4 (8) %	44 (19.3) %
	Mean DeltaNaM1 (SD)	0.110 (n.a.)	0.043 (0.042)	n.s.	n.s.	0.067 (0.038)	0.036 (0.049)
	Mean CrNaM1 (SD)	28.2 (n.a.) %	13.2 (12) %	15.8 (12.7) %	7.1 (n.a.) %	21.8 (7.8) %	9.4 (13) %

T—thoracic, L—lumbar, TL—thoracolumbar, SD—single standard deviation; n.s.—not significant in multivariate analysis, n.a.—not applicable (*n* = 1); (+) means “present”, (−) means “not present”.

**Table 5:** Combined brace parameter effects on (major) curve.

ASP		TS(+)	TS(−)	PHTP(+)	PHTP(−)
yes	Mean DeltaCobb1 (SD)	9.9 (8.3) °	9 (8.7) °	9.6 (8.6) °	8.2 (6.6) °
	Mean CrCobb1 (SD)	29.7 (19.3) %	31.7 (27.8) %	29.7 (23.1) %	40.9 (35.1) %
	Mean DeltaNaM1 (SD)	n.s.	n.s.	0.024 (0.046)	0.055 (0.035)
	Mean CrNaM1 (SD)	10.1 (12) %	7.2 (17) %	7.4 (14.8) %	19.3 (8) %
no	Mean DeltaCobb1 (SD)	10.6 (6.4) °	11.8 (5.9) °	11.4 (6.3) °	8.4 (6) °
	Mean CrCobb1 (SD)	33.9 (20.6) %	44.5 (25) %	38 (22) %	31.5 (25.4) %
	Mean DeltaNaM1 (SD)	n.s.	n.s.	0.020 (0.043)	0.004 (0.032)
	Mean CrNaM1 (SD)	5.7 (15.9) %	6.2 (13.5) %	6.5 (15.4) %	1.3 (12.9) %

SD—single standard deviation, n.s.—not significant in multivariate analysis.

## Data Availability

Data can be requested from the authors.
